# Reproducibility of left ventricular blood flow kinetic energy measured by four-dimensional flow CMR

**DOI:** 10.1186/s13104-021-05697-3

**Published:** 2021-07-27

**Authors:** Ciaran Grafton-Clarke, Saul Crandon, Jos J. M. Westenberg, Peter P. Swoboda, John P. Greenwood, Rob J. van der Geest, Andrew J. Swift, Vassilios S. Vassiliou, Sven Plein, Pankaj Garg

**Affiliations:** 1 George Davies Centre, School of Medicine, University of Leicester, Lancaster Road, Leicester, LE1 7HA UK; 2 Leeds Institute of Cardiovascular and Metabolic Medicine, University of Leeds, Leeds, UK; 3 Department of Radiology, Leiden University Medical Center, Leiden, The Netherlands; 4 Department of Infection, Immunity & Cardiovascular Disease, University of Sheffield, Sheffield, UK; 5 Norwich Medical School, University of East Anglia, Norwich, UK

**Keywords:** 4D flow CMR, Haemodynamics, Flow quantification, Reproducibility analysis

## Abstract

**Objectives:**

Four-dimensional flow CMR allows for a comprehensive assessment of the blood flow kinetic energy of the ventricles of the heart. In comparison to standard two-dimensional image acquisition, 4D flow CMR is felt to offer superior reproducibility, which is important when repeated examinations may be required. The objective was to evaluate the inter-observer and intra-observer reproducibility of blood flow kinetic energy assessment using 4D flow of the left ventricle in 20 healthy volunteers across two centres in the United Kingdom and the Netherlands.

**Data description:**

This dataset contains 4D flow CMR blood flow kinetic energy data for 20 healthy volunteers with no known cardiovascular disease. Presented is kinetic energy data for the entire cardiac cycle (global), the systolic and diastolic components, in addition to blood flow kinetic energy for both early and late diastolic filling. This data is available for reuse and would be valuable in supporting other research, such as allowing for larger sample sizes with more statistical power for further analysis of these variables.

## Objective

Quantitative cardiovascular magnetic resonance (CMR) imaging can provide a wealth of information to distinguish health from disease [1]. Four-dimensional flow (4D flow) CMR allows for a comprehensive assessment of the blood flow kinetic energy (KE) of the left ventricle (LV) [2]. Assessment of LV KE by 4D flow CMR is thought to offer superior reproducibility compared to standard two-dimensional phase contrast acquisition [3]. In our publication, for which this dataset corresponds to [4], we set out to answer questions we felt to be fundamental in evaluating the diagnostic utility of 4D flow CMR. First, we determined the normal ranges of LV KE values across the spectrum of age, which is an important step in differentiating between healthy and diseased states. Second, given the established association between standard 2D parameters of diastolic function and the myocardial stiffening accompanying the ageing process [5], we investigated the association of LV blood flow KE using 4D flow CMR with 2D mitral inflow and myocardial tissue velocities by CMR. Third, we assessed inter-observer and intra-observer reproducibility of 4D flow CMR LV KE assessments. Reproducibility is a prerequisite for any investigatory technique where repeated examinations may be required.

Reproducibility in cardiac MRI research is a pervasive issue across the field, which impacts the translational pathway [6]. Given the relative infancy of research within 4D flow CMR and the accepted potential for this imaging technique to propel our understanding of cardiovascular disease, it becomes of utmost importance to establish repeatability of the various aspects of the techniques, ideally across multiple centres. We hope the publication of our data will offer further opportunities to generate research which contributes to our growing understanding of the diagnostic utility of 4D flow CMR techniques. Our data will be of particular use to research groups incorporating reproducibility analysis within validation studies, and is available for reuse and assimilation within quantitative syntheses.

## Data description

The dataset includes the LV blood flow KE parameters of twenty healthy adult volunteers void of any known cardiovascular disease, collected from two sites in the United Kingdom (Leeds) and the Netherlands (Leiden). CMR was performed on a dedicated cardiovascular 1.5 Tesla Phillips Ingenia system equipped with a 28-channel coil and Phillips dStream digital broadband MR architecture technology. The data was acquired following analysis of CMR images using dedicated 4D flow research software (Mass; Version 2017EXP, Leiden University Medical Center, Leiden, The Netherlands). After acquiring images, advanced 4D flow CMR techniques were applied to determine the kinetic energy (KE) for each phase of the cardiac cycle, following which all KE parameters were normalized to the LV end-diastolic volume, and reported in standard units (μJ/ml).

The dataset is composed of two parts. First, inter-observer reproducibility. This refers to two separate operators analysing the twenty cases independent of each other. Second, intra-observer reproducibility. This refers to the same operator analysing the same ten cases on two separate occasions, with a three-month interval between. The data presented is the endpoint of a range of software-delivered processing of the images, which is beyond the scope of this report. Further detail on the 4D LV KE mapping can be found within our primary publication on this topic [4]. The reproducibility aspect presented within the dataset is the result of assessor contouring of the short-axis LV cine volumetric stacks prior to automation and manual readjustment. The number of cases for both inter- and intra-observer reproducibility exceeds the minimum number required as per power calculations based upon previous KE studies. This dataset should serve as a valuable benchmark for other research groups in establishing the required sample sizes to ensure adequate statistical power within CMR trials, based on the reproducibility outcomes identified from this cohort. This dataset, when analysed using the appropriate correlation statistical tests, demonstrated excellent concordance in global KE parameters for both inter and intra-observer reproducibility analyses.

For each case within the two reproducibility analyses, we include data for: global LV KE (an average KE of the LV flow for the complete cardiac cycle), systolic KE (the average KE of the LV flow during systole), diastolic KE (the average KE of the LV flow during diastole), peak E-wave KE (the peak KE of the LV flow during early diastolic filling), and peak A-wave KE (the peak KE of the LV flow during late diastolic filling).

## Limitations

The temporal resolution of 4D flow is 40 ms (ms), which may impact the precision of KE assessments. This is an issue characteristic of 4D flow analyses, rather than a limitation unique to our dataset. 4D flow was acquired during free-breathing, for which issues relating to heart rate may have impacted on the time-varying flow characteristics, which could not be corrected for. There are no other immediate limitations of this dataset*.*

## Data Availability

The data described in this Data note can be freely and openly accessed on Harvard Dataverse under https://doi.org/10.7910/DVN/JMTN02. Please see Table

**Table 1:** Overview of data files/data sets

Label	Name of data file/data set	File types (file extension)	Data repository and identifier (DOI or accession number)
Data file 1	Datasheet (inter-observer)	MS excel file (.xlsx)	Harvard Dataverse [7] (https://doi.org/10.7910/DVN/JMTN02)
Data file 2	Datasheet (intra-observer)	MS excel file (.xlsx)	Harvard Dataverse [7] (https://doi.org/10.7910/DVN/JMTN02)

1 and references for details and links to the data [7]. Overview of data files/data sets

## Abbreviations

2D: 2 Dimensional
4D-Flow: 4-Dimensional flow
CMR: Cardiovascular magnetic resonance
KE: Kinetic energy
LV: Left ventricle
